# Prion Strain-Specific Structure and Pathology: A View from the Perspective of Glycobiology

**DOI:** 10.3390/v10120723

**Published:** 2018-12-18

**Authors:** Ilia V. Baskakov, Elizaveta Katorcha, Natallia Makarava

**Affiliations:** 1 Center for Biomedical Engineering and Technology, University of Maryland School of Medicine, Baltimore, MA 21201, USA; elizaveta.katorcha@gmail.com (E.K.); nmakarava@som.umaryland.edu (N.M.); 2 Department of Anatomy and Neurobiology, University of Maryland School of Medicine, Baltimore, MA 21201, USA

**Keywords:** prions, prion disease, prion strains, N-linked glycans, glycosylation, sialic acid, sialylation

## Abstract

Prion diseases display multiple disease phenotypes characterized by diverse clinical symptoms, different brain regions affected by the disease, distinct cell tropism and diverse PrP^Sc^ deposition patterns. The diversity of disease phenotypes within the same host is attributed to the ability of PrP^C^ to acquire multiple, alternative, conformationally distinct, self-replicating PrP^Sc^ states referred to as prion strains or subtypes. Structural diversity of PrP^Sc^ strains has been well documented, yet the question of how different PrP^Sc^ structures elicit multiple disease phenotypes remains poorly understood. The current article reviews emerging evidence suggesting that carbohydrates in the form of sialylated N-linked glycans, which are a constitutive part of PrP^Sc^, are important players in defining strain-specific structures and disease phenotypes. This article introduces a new hypothesis, according to which individual strain-specific PrP^Sc^ structures govern selection of PrP^C^ sialoglycoforms that form strain-specific patterns of carbohydrate epitopes on PrP^Sc^ surface and contribute to defining the disease phenotype and outcomes.

## 1. Introduction

Prion diseases or transmissible spongiform encephalopathies represent a class of lethal, transmissible neurodegenerative disorders of humans and animals [1,2]. Prions or PrP^Sc^ are proteinaceous infectious agents that consist of misfolded, self-replicating states of a sialoglycoprotein called the prion protein or PrP^C^. Prions spread between organisms or from cell to cell by recruiting host-encoded PrP^C^ and replicating their disease-specific misfolded structures via a template-assisted mechanism [3]. Prion diseases display multiple disease phenotypes characterized by diverse clinical symptoms, different brain regions affected by the disease, distinct cell tropism and diverse PrP^Sc^ deposition patterns [4]. The diversity of disease phenotypes within the same host is attributed to the ability of PrP^C^ to acquire multiple, alternative, conformationally distinct, self-replicating PrP^Sc^ states referred to as prion strains or subtypes [5,6,7,8,9,10]. Structural diversity of PrP^Sc^ strains has been well documented [11,12,13], yet the question of how different PrP^Sc^ structures elicit multiple disease phenotypes remains poorly understood.

PrP^C^ is post-translationally modified with a glycophosphatidylinositol (GPI) anchor and up to two N-linked glycans [14,15,16,17]. These modifications are carried over upon conversion of PrP^C^ into PrP^Sc^ [14,16,17,18,19]. PrP^C^ expressed in a cell exhibit astonishing heterogeneity, which is attributed to variations in the structure and composition of N-glycans with more than 400 different PrP^C^ glycoforms described [19,20,21]. Because each of the two N-glycans can carry up to five and possibly even more negatively charged sialic acid residues [19,20,21], PrP^C^ molecules also display a dramatic range of pIs or net charges at physiological pH [22,23]. Considering their bulky size, a broad range of negative charges and extreme structural heterogeneity, we were interested to learn whether N-glycans played a role in defining strain-specific PrP^Sc^ structural and pathological features.

## 2. N-glycans Are Exposed on a Surface of PrP^Sc^

**Figure 1:**
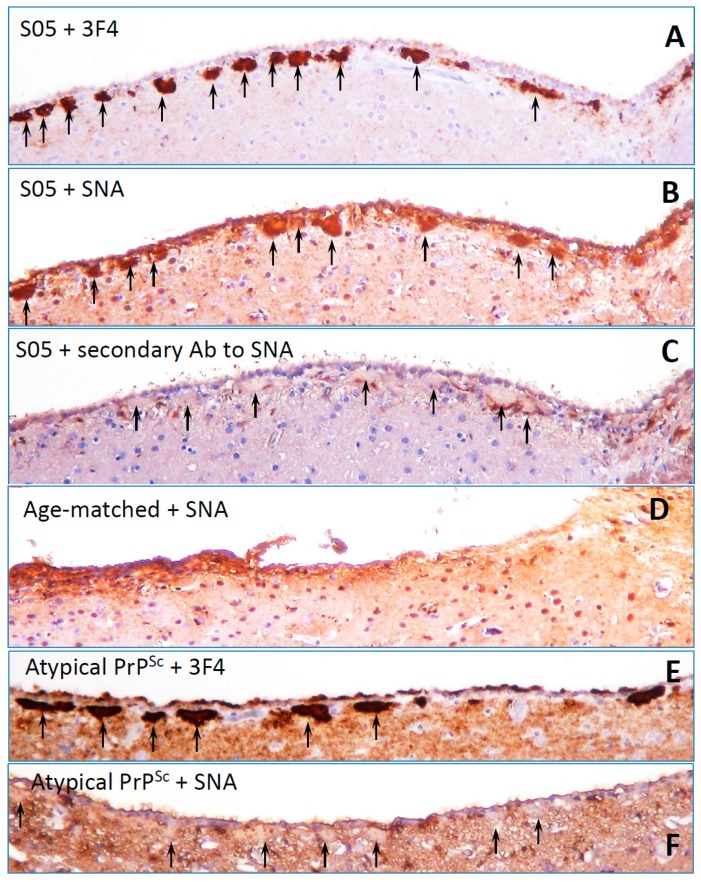
Staining of PrP^Sc^ plaques with SNA lectin. Images of SO5 PrP^Sc^ plaques (**A**–**C**) or atypical PrP^Sc^ plaques (**E**,**F**) in hamster brains stained with anti-PrP 3F4 antibody (**A**,**E**), SNA lectin (**B**,**F**), or secondary antibody used for SNA staining as negative control (**C**). Plaques are shown by arrows. Staining of normal age-matched control with SNA lectin is shown in (**D**). Brains fixed in 10% neutral buffered formalin were treated with 95% formic acid for 1 h before embedding in paraffin wax and sectioning into 4 µm sections. After a standard rehydration procedure, slides were submerged in 10 mM tri-sodium citrate buffer, pH 6.0, boiled for 5 min by microwaving at 20% power, and cooled for 1 h before proceeding with lectin staining. Incubation in 3% hydrogen peroxide in methanol for 20 min was used to remove endogenous peroxidase activity. After 5 min wash in running water, slides were incubated for 1 h with 5 µg/mL biotin-labeled elderberry bark lectin (SNA, Vector laboratories, Burlingame, CA) diluted in lectin buffer, pH 7.6 (50 mM Tris, 150 mM NaCl, 1 mM MgCl2, 0.75 mM CaCl2). Following triple 5 min wash in lectin buffer, the slides were incubated for 30 min in 5 µg/mL horse radish peroxidase-labeled streptavidin (Thermo Fisher scientific, Waltham, MA), then again washed three times with lectin buffer, and developed with 3,3’ Diaminobenzidine (DAB) Quanto chromogen and substrate (VWR, Radnor, PA).

Molecular models of PrP^Sc^ particles proposed in the past assumed that the N-glycans are directed outwards [24,25,26] exposing terminal sialic acid residues on the surface of PrP^Sc^ particles. For testing whether N-glycans are indeed directed outwards of PrP^Sc^ particles, brain slices from scrapie-infected animals were stained using *Sambucus Nigra* lectin (SNA). Animals infected with the S05 strain were selected, as this strain is characterized by deposition of large PrP^Sc^ plaques [27,28]. In PrP^C^ and PrP^Sc^, sialic acid residues are attached to galactose via α2-3 or α2-6 linkages with the majority being linked via α2-6 [20,21,29]. SNA lectin was used for staining, because of its specificity for sialic acid residues attached via α2-6 linkages. As expected, PrP^Sc^ plaques showed intense staining with SNA (Figure 1A–D). In fact, higher intensity of PrP^Sc^ staining relative to that of the brain sialoglycocalix suggests that the density of sialylation on PrP^Sc^ surface exceeds that of sialoglycocalyx. This result is consistent with the previous study that report intense staining of PrP^Sc^ plaques by Alcian Blue, a polyvalent basic dye which is used for staining of sialylated glycocalyx [30]. High intensity of straining with both SNA and Alcian Blue illustrate a high density of sialylation on PrP^Sc^ surface.

## 3. Structural Constraints Imposed by N-glycans

Several structures that exhibit diverse PrP folding patterns have been considered in recent years to model the structure of PrP^Sc^ (reviewed in [26]). They include parallel in-register β-structure and two-, three- or four-rung β-solenoids [25,31,32,33,34]. To build a realistic molecular model of PrP^Sc^, our knowledge about PrP^Sc^ glycosylation status, size of the glycans and their charge should be considered. Which of the proposed models can accommodate N-linked glycans?

For addressing this question, a tri-antennary glycan with a size average of those found in PrP sialoglycoforms was used for modeling PrP^Sc^ [35]. In in-register parallel β-sheet structure, the glycans of neighboring PrP molecules have to be spaced at a distance of 4.7 Å that brings them into substantial spatial interference that precludes such arrangements [35]. Considerable spatial overlap between glycans still exists in a two-rung solenoid that separates glycans at a distance of 2 × 4.7 Å [35]. However, three-rung solenoids permit recruitment with minimal interference, whereas four-rung solenoid can accommodate diglycosylated PrP molecules without interference [35]. This modeling supports the hypothesis that N-glycans limit the diversity of folding patterns accessible to glycosylated PrP^C^.

## 4. Two Alternative Views on Involvement of N-glycans

Prion strains display strain-specific ratios of di-, mono- and unglycosylated glycoforms, which are often considered to be among the features defining strain^.^ identity (reviewed in [36]). Toward answering the question of whether glycans are important in defining strain-specific PrP^Sc^ structures, two alternative views could be taken into consideration. According to one view, prion strains can partially overcome constraints imposed by glycans by selectively recruiting mono- and un-glycosylated PrP^C^ glycoforms at the expense of diglycosylated glycoforms. Only those PrP^C^ glycoforms could be successfully recruited that fit into strain-specific PrP^Sc^ structures. An alternative view proposes that recruitment is not selective; glycoforms are recruited proportionally to their relative representations in a pool of PrP^C^ molecules. If this is the case, the spectrum of PrP^Sc^ structures would be limited to those that can accommodate a high percentage of diglycosylated glycoforms, since diglycosylated glycoforms dominate the pool of PrP^C^ molecules.

**Figure 2:**
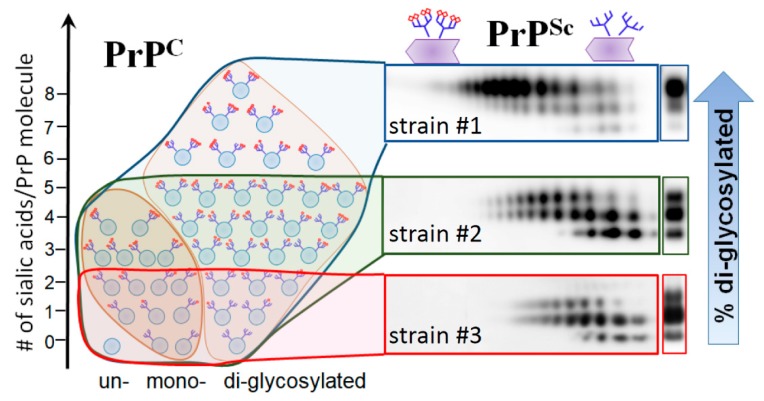
Schematic diagram illustrating selective recruitment of PrP^C^ sialoglycoforms in a strain-specific manner according to the PrP^C^ sialylation status. The left panel shows distribution of PrP^C^ molecules according to their glycosylation status (in horizontal dimension) and sialylation status (in vertical dimension) ranging from non-sialylated to highly sialylated molecules. PrP^C^ molecules are shown as blue circles and sialic acid residues as red diamonds. The panels on the right show 2D Western blots of three prion strains with different recruitment selectivity. While 263K (strain #1) recruits PrP^C^ sialoglycoforms without strong preferences, hypersialylated PrP^C^ molecules are preferentially excluded from RML (strain #2) and excluded even stronger from atypical PrP^Sc^ (strain #3). As a result of strain-specific exclusion of highly sialylated PrP^C^, ratios of glycoforms within PrP^Sc^ shift toward mono- and unglycosylated glycoform, as illustrated by corresponding 1D Western blots. Adapted from Baskakov and Katorcha 2016 [35].

To answer the question of whether prion strains recruit PrP^C^ sialoglycoforms selectively, we analyze the composition of sialoglycoforms within PrP^Sc^ using 2D gels [37,38]. Because N-linked glycans carry negatively charged sialic acid residues, the sialoglycoforms can be separated in a horizontal dimension of 2D according to their charge [35,39]. For the 2D, PrP^Sc^ was denatured into monomers first, and then monomers were separated according to their sialylation status in horizontal dimension and their glycosylation status in vertical dimension. Then, 2D analysis of multiple strains from two hosts revealed that PrP^Sc^ strains exhibit a broad range of strain-specific selectivity with respect to PrP^C^ sialoglycoforms (Figure 2) [37]. Consistent with the first view, a group of strains shows strong preferences, as they excluded highly sialylated molecules as well as diglycosylated molecules (Figure 2, represented by strain #2). At the same time, in support of the second view, a group of strains did not display any preferences with respect to glycosylation or sialylation status (Figure 2, represented by strain #1). In addition, atypical PrP^Sc^ of synthetic origin was highly selective [28,40], as it excluded most of the diglycosylated and also highly and moderately sialylated PrP^C^ molecules (Figure 2, strain #3).

**Figure 3:**
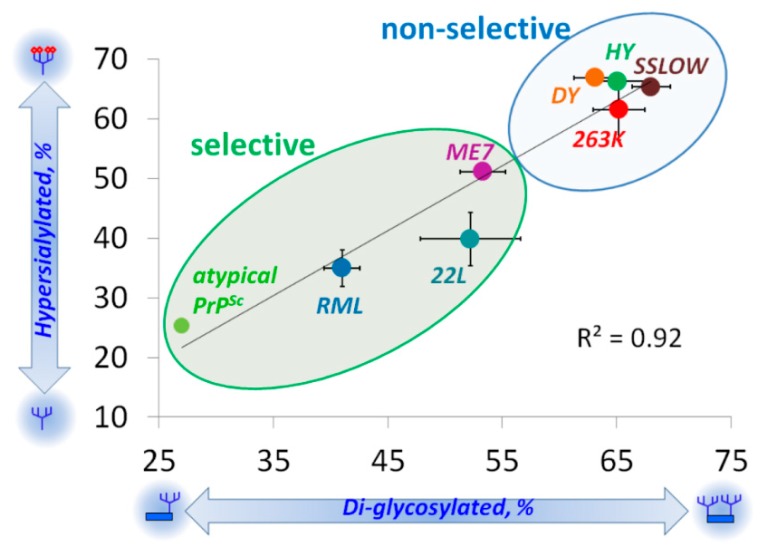
Correlation between strain-specific sialylation status and glycoform ratio. Strain-specific percentages of diglycosylated glycoforms plotted as a function of strain-specific percentage of hypersialylated glycoforms within PrP^Sc^. Mean and standard deviations are shown (n = 3 animals). Black solid line shows the result of linear fitting of the percent of diglycosylated as a function of the percent of hypersialylated for brain-derived PrP^Sc^. Adapted from Katorcha et al. 2015 [37].

Analysis across all strains examined revealed a great correlation between the glycosylation and sialylation status within PrP^Sc^ (Figure 3) [37]. This analysis also demonstrated a broad range of selectivity displayed by prion strains in recruiting PrP^C^ sialoglycoforms, ranging from non-selective to highly selective (Figure 3). Notably, for the group of non-selective strains, the composition of sialoglycoforms within PrP^Sc^ was very similar to that of PrP^C^.

## 5. Relationship between Strain-Specific Structure and Selectivity for PrP^C^ Sialoglycoforms

**Figure 4:**
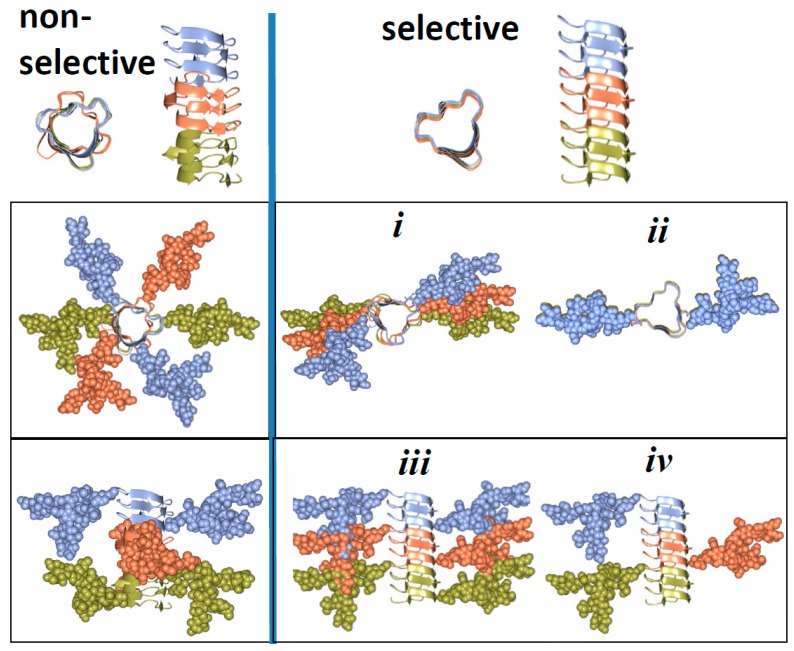
Schematic diagram illustrating differences in quaternary assembly between non-selective (left panels) and selective (right panels) strains. It is proposed that non-selective strains can accommodate diglycosylated sialoglycoforms because of rotation between neighboring PrP molecules that allows spatial separation of glycans and reduces electrostatic repulsion. In selective strains, the rotation between neighboring PrP molecules is very small (*i*) or absent (*ii*). Recruitment of diglycosylated molecules by selective strains would lead to spatial interference and electrostatic repulsion between glycans (*iii*). Negative selection of diglycosylated molecules helps to minimize spatial and electrostatic interference between glycans (*iv*). While the same principles are applicable to both three- and four-rung solenoid structure, for simplicity of presentation only three-rung solenoid structures are shown here.

Here we propose that a broad range of selectivity with respect to recruiting PrP^C^ sialoglycoforms expressed by prion strains could be attributed to strain-specific variations in quaternary structure (i.e., assembly of PrP monomers into multimers within PrP^Sc^) and achieved in the absence of significant strain-specific variations in folding pattern. Specifically, we propose that for non-selective strains, there is a considerable twist or rotation between neighboring PrP molecules assembled in PrP^Sc^ particles (Figure 4). Owing to such rotations, glycans extend into different directions avoiding spatial interference and minimizing electrostatic repulsion between their sialic acid residues. In strains that select against diglycosylated and highly sialylated PrP^C^, the twist between neighboring PrP molecules is very small or absent (Figure 4). As a result, such a mode of assembly creates spatial and electrostatic interference between glycans limiting recruitment of diglycosylated and highly sialylated PrP^C^. The three- or four-rung solenoid models offer the best opportunity for accommodating both selective and non-selective strains. The proposed arrangement of N-linked glycans and sialic acid residues in selective and non-selective strains (Figure 4) could be used to distinguish these strains experimentally, based on their ultrastructure or fibril morphology. If our hypothesis is correct, non-selective strains are expected to display twisted fibril morphologies, whereas selective strains should show less twisted or non-twisted morphologies.

Previous studies employed differentially glycosylated PrP^C^ molecules and Protein Misfolding Cyclic Amplification (PMCA) to explore the role of N-linked glycans in prion replication [41]. Consistent with the model presented in Figure 4, unglycosylated PrP^C^ molecules were required for propagating the RML strain [41], which belongs to the group of selective strains (Figure 3). Propagation of the Sc237 strain, which has the same origin as the 263K stain and presumably belongs to the group of non-selective strains (Figure 3), relied on diglycosylated PrP^C^ molecules [41]. The fact that diglycosylated PrP^C^ molecules were required for replication of the Sc237 strain suggests that N-linked glycans might play a role in stabilizing multimeric structures of non-selective strains via intermolecular interactions.

## 6. Role of N-glycans in Maintaining High Fidelity of Prion Replication

Prions replicate with high fidelity when transmitted within the same host. Do N-glycans play a role in preserving high fidelity of prion replication? Considering that N-glycans limit diversity of folding patterns accessible to PrP^C^, removal of PrP^C^ glycans is expected to impair fidelity of replication. Eliminating the entire glycans from donor PrP^Sc^ did not affect strain-specific neurotropism upon transmission of two strains, RML or 301C, to wild type host with unaltered glycosylation status of PrP^C^ [42]. However, this was not the case when glycosylation status of PrP^C^ expressed by a host was altered. Transmission of prions to hosts expressing PrP^C^ deficient in one or both N-glycans was found to change strain-specific characteristics of the 79A strain, yet did not affect considerably strain-specific properties of ME7 or 301C [43]. Moreover, glycosylation of the host PrP^C^ was found to have a significant impact on the transmission of prions between different species [44]. These studies support the view that loss of structural constraints imposed by PrP^C^ glycans might indeed impair fidelity of prion replication to the extent that, in some cases, result in changes of strain properties and disease phenotype.

## 7. Role of Sialic Acid Residues and Electrostatic Repulsions in Prion Replication/Strain-Specific Properties

**Figure 5:**
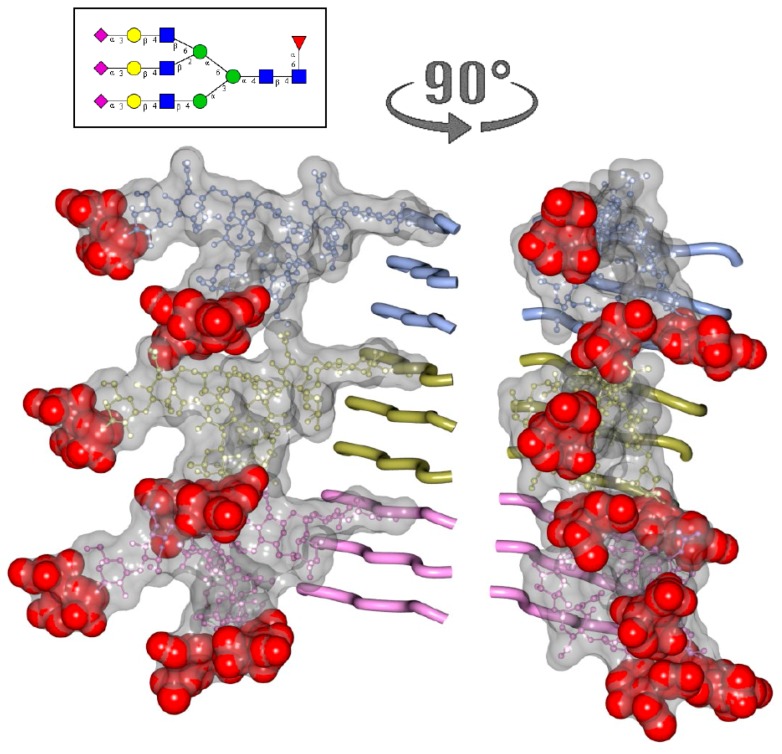
Modeling of N-linked glycans in PrP^Sc^ consisting of three-rung solenoid. Two views of three-rung solenoid structures carrying tri-antennary N-glycans. Polypeptide chains are represented in the tube form, whereas N-glycans are represented in the ball-and-stick form. Each PrP molecule with corresponding N-glycan is of a different color. Sialic acid residues are colored in red. The structure of a tri-antennary N-linked glycan (shown in inset) was taken from PDB entry 3QUM, a crystal structure of human prostate specific antigen (PSA) [45]. Both calculations of electrostatic surfaces and generation of images were performed with CCP4MG software. The model based on three-rung solenoid structure is shown here for simplicity of presentation and should not be considered as preferable over the four-rung solenoid model.

Assuming that PrP^Sc^ adapts three- or four-rung solenoid folding patterns, sialylation of N-linked glycans would result in a highly dense negative charge on PrP^Sc^ surface (Figure 5). Does electrostatic repulsion between sialic acid residues impose constraints on PrP^Sc^ surfaces that affects prion replication rate? If electrostatic repulsion is indeed substantial, one might expect that removing sialic acid residues from PrP^C^ should facilitate PrP^C^ recruitment and accelerate replication rates of PrP^Sc^. For testing whether this is the case, amplification rates of several prion strains were examined using PMCA conducted in PrP^C^ substrate desialylated by neuraminidase treatment, and in normal non-treated substrates [22,37]. As predicted by the above hypothesis, a significant increase in replication rates was observed in desialylated relative to normal substrates for all strains tested [22,37]. Moreover, an increase in replication rates was found to be strain-specific ranging from 10-fold to ~ 10^6^-fold [22,37]. The fact that removing sialic acids boosts replication rates in a strain-specific manner suggests that PrP^Sc^ surfaces are decorated by sialic acid in strain-specific patterns generating strain-specific density of negative charges.

As discussed above, a strain-specific glycoform ratio is a result of negative selection of diglycosylated sialoglycoforms carrying bulky, highly charged glycans. Is it electrostatic repulsion between sialic acid residues or spatial interference between bulky glycans that imposes constraints and limits recruitment of diglycosylated glycforms? To answer this question, glycoform ratios were analyzed in PrP^Sc^ generated from desialylated substrate in PMCA. In PMCA-derived PrP^Sc^ produced from desialylated substrate, the relative ratio of glycoforms shifted to predominantly diglycosylated regardless of the glycosylation status of strains used for seeding [37]. In fact, upon replication in desialylated substrate, prion strains lost their strain-specific glycoform ratios that become very similar to the glycoform ratio of PrP^C^ [37]. In summary, these experiments revealed that negative selection of diglycosylated glycoforms is abolished, when sialic acid residues are removed. As such, the sialylation level appears to be even more important than the size of glycans in imposing constraints and defining strain-specific glycoform ratios.

## 8. Surface Carbohydrate Epitopes of PrP^Sc^ as Molecular Cues for CNS

Under a normal replication environment, prion strains maintain their strain-specific ratios of glycoforms that range from predominantly un- and mono-glycosylated to highly diglycosylated (reviewed in [36]). Differences in glycosylation status, which is presumably attributed to strain-specific structural constraints, suggest that each strain can accommodate only a certain number of sialylic acid residues per PrP molecule on PrP^Sc^ surface yielding a strain-specific density of negative charges. Moreover, as a result of selective recruitment, each strain is likely to display an individual, strain-specific pattern of carbohydrate epitopes on PrP^Sc^ surface. One might speculate that strain-specific patterns of carbohydrate epitopes will define the range of potential PrP^Sc^-binding partners interacting with PrP^Sc^ giving rise to individual, strain-specific biological features. Several protein families including siglecs, selectins, galectins, complement system components, mannose receptors and asialoglycoprotein receptors recognize carbohydrate groups and are involved in an innate immune response [46,47]. Because the majority of carbohydrate-binding molecules have multivalent binding sites, the strength and selectivity of binding is not only dependent on the composition of functional carbohydrate epitopes but also on their density and specific configuration of carbohydrate groups.

**Figure 6:**
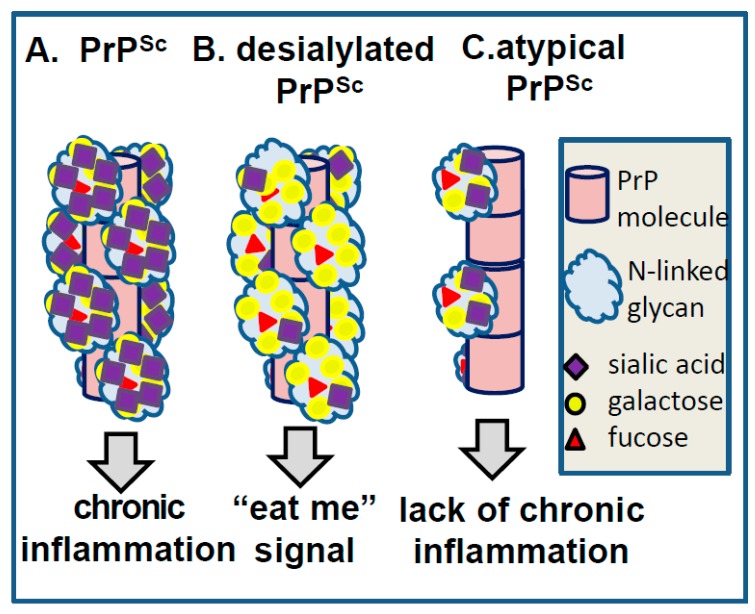
Schematic representation of the hypothesis proposing that carbohydrate epitopes on PrP^Sc^ surface determine response of glia. A. High density of glycans with terminal sialylation leads to chronic neuroinflammation. B. Desialylation of PrP^Sc^ that results in a high density of exposed galactose triggers “eat me” signal in glia. C. Atypical PrP^Sc^ has low density of glycosylation and sialylation, similar to those of sialoglycocalyx. Atypical PrP^Sc^ does not trigger “eat me” signal or chronic neuroinflammation.

Below, we discuss specific examples illustrating involvement of carbohydrate epitopes on PrP^Sc^ surface in triggering CNS response and determining the fate of prions (Figure 6). Our recent studies revealed that desialylation of N-linked glycans of PrP^Sc^ undermines survival of prions in vivo. On the surface of a mammalian cell, sialoglycocalyx acts as a part of a “self-associated molecular pattern” helping the innate immune system to recognize “self” from “altered self” or “non-self” [48,49]. Removal of sialic acids from cell surface glycans exposes galactose residues that generate “eat me” signals for professional and non-professional macrophages, for example, stimulating clearance of erythrocytes or platelets by Kupffer cells [50,51], or phagocytosis of neurons by microglia [52,53,54]. Maintaining sufficient sialylation levels appears to be as important for prion survival as it is for preserving healthy mammalian cells (Figure 6A,B). Our recent studies demonstrated that donor PrP^Sc^ with reduced sialylation levels did not induce prion disease in animals after intracranial or intraperitoneal inoculations [22,55,56] (Figure 6A,B). Moreover, animals inoculated with PrP^Sc^ with reduced sialylation levels were found to be free of prions for their life-time [55,56]. Upon peripheral exposure, prions are known to be first uptaken by dendritic cells, monocytes and B lymphocytes [57,58], which spread prions through the body before they are sequestered by secondary lymphoid organs (SLOs) [59,60,61,62]. Prions replicate and are accumulated in SLOs prior to their invasion of CNS [59,60,61,62,63,64]. Sialylation of PrP^Sc^ was found to be critical for its trafficking and colonization of SLOs [56]. PrP^Sc^ with reduced sialylation was found in the liver instead of SLOs. Yet, PrP^Sc^ that colonizes SLOs was shown to be subject to enhanced post-conversion sialylation, a process that might provide extra protection from the innate immune system [38]. These studies suggested that sialylation protects PrP^Sc^ against clearance and appears to be critical in defining the fate of prion infection. Consistent with this hypothesis, our recent work revealed that PrP^Sc^ can directly trigger proinflammatory response in microglia with a degree of response found to be determined by the degree of sialylation of PrP^Sc^ [65].

Another example that might shed light on the relationship between PrP^Sc^ molecular features and neuroinflammation deals with a unique PrP^Sc^-like state that has substantially reduced both glycosylation and sialylation levels (will be referred to as atypical PrP^Sc^) [27,40,66]. Atypical PrP^Sc^ is a self-replicating transmissible state [28,67,68], which accumulates in the form of small synaptic deposits and large plaques [66]. Yet, it is a clinically silent state that does not cause neuronal death, pathological lesions or any detectible clinical signs of the prion diseases [27,28,40,66,67]. Remarkably, atypical PrP^Sc^ recruits mono- and un-glycosylated PrP^C^ of a host, while strongly excluding di-glycosylated and sialylated PrP^C^ molecules [28] (strain #3 in Figure 2, and Figure 3). In fact, out of ten strains tested, atypical PrP^Sc^ had the lowest glycosylation and sialylation status [28,37]. Staining with SNA confirmed that sialylation levels of atypical PrP^Sc^ is significantly lower relative to those of conventional PrP^Sc^ or brain sialoglycocalyx (Figure 1E,F). Yet, unlike desialylated PrP^Sc^, atypical PrP^Sc^ does not expose terminal galactose at high density, which is considered to be an “eat me” signal (Figure 6). As a result, it replicates and accumulates steadily in CNS. Atypical PrP^Sc^ lacks toxicity and does not case neuroinflammation [66], features that appear to be attributed to its unique glycosylation and sialylation status.

## 9. Concluding Remarks

N-glycans are exposed on the PrP^Sc^ surface and impose considerable structural constraints to PrP^Sc^ assembly due to their bulky size and electrostatic repulsion between sialic acid residues. To overcome these restraints, prion strains exhibit a wide range of selectivity in recruiting PrP^C^ sialoglycoforms. While some strains recruit sialoglycoforms proportionally to their representation in PrP^C^, others avoid diglycosylated and highly sialylated PrP^C^ glycofoms. We propose that strain-specific selectivity in recruiting PrP^C^ sialoglycoforms reports on strain-specific differences in PrP^Sc^ quaternary structures and has to be taken into consideration when building realistic PrP^Sc^ models. Moreover, we hypothesize that as a result of strain-specific selection, N-linked glycans form individual strain-specific patterns of functional carbohydrate epitopes on PrP^Sc^ surfaces and elicit strain-specific responses in CNS. It has been well established that the innate immune system senses terminal carbohydrate groups including galactose and sialic acid residues, which can serve as molecular cues and trigger diverse response programs by glia. Consistent with previous studies on self-associated and pathogen-associated molecular patterns, our work revealed that desialylation of PrP^Sc^ undermines survival of prions in vivo. At the same time, recruiting PrP^C^ with low sialylation levels speeds up PrP^Sc^ replication, which could be beneficial to prion spread. Therefore, evolution of prion strains might represent a delicate balance between recruiting of highly sialylated glycoforms and generating sufficient density of sialylation for avoiding an “eat-me” response by glia, yet limiting extremely heavily sialylated glycoforms for enabling efficient prion replication. It remains to be tested in future studies whether carbohydrate epitopes on PrP^Sc^ surface trigger responses in the CNS in a strain-specific manner.
